# Long-Term Outcomes of Patients with Acute Cholecystitis after Successful Percutaneous Cholecystostomy Treatment and the Risk Factors for Recurrence: A Decade Experience at a Single Center

**DOI:** 10.1371/journal.pone.0148017

**Published:** 2016-01-28

**Authors:** Chih-Hung Wang, Cheng-Yi Wu, Justin Cheng-Ta Yang, Wan-Ching Lien, Hsiu-Po Wang, Kao-Lang Liu, Yao-Ming Wu, Shyr-Chyr Chen

**Affiliations:** 1 Department of Emergency Medicine, National Taiwan University and National Taiwan University Hospital, Taipei, Taiwan; 2 Department of Medical Imaging, National Taiwan University and National Taiwan University Hospital, Taipei, Taiwan; 3 National Taiwan University Hospital, Hsin-Chu Branch and National Taiwan University, Hsinchu City, Taiwan; 4 Department of Internal Medicine, National Taiwan University and National Taiwan University Hospital, Taipei, Taiwan; 5 Department of Surgery, National Taiwan University and National Taiwan University Hospital, Taipei, Taiwan; Kaohsiung Chang Gung Memorial Hospital, TAIWAN

## Abstract

**Background:**

Percutaneous cholecystostomy tube (PCT) has been effectively used for the treatment of acute cholecystitis (AC) for patients unsuitable for early cholecystectomy. This retrospective study investigated the recurrence rate after successful PCT treatment and factors associated with recurrence.

**Methods:**

We reviewed patients treated with PCT for AC from October 2004 through December 2013. Patients with successful PCT treatment were those who were free from persistent PCT drainage. We used multivariable logistic regression analysis sequentially to identify factors associated with each outcome.

**Results:**

The study included 184 patients (mean age: 70.1 years). The average duration for parenteral antibiotics was 14.4 days and 20.0 days for PCT drainage. The one-year recurrence rate was 9.2% (17/184) with most recurrences occurring within two months (6.5%, 12/184) of the procedure. Complicated cholecystitis (odds ratio [OR]: 4.67; 95% confidence interval [CI]: 1.44–15.70; *P* = 0.01) and PCT drainage duration >32 days (OR: 4.92; 95% CI: 1.03–23.53; *P* = 0.05) positively correlated with one-year recurrence; parenteral antibiotics duration >10 days (OR: 0.21; 95% CI: 0.05–0.68; *P* = 0.01) was inversely associated with one-year recurrence.

**Conclusions:**

The recurrence rate was low for patients after successful PCT treatment. Predictors for recurrence included the severity of initial AC and subsequently provided treatments.

## Introduction

Acute cholecystitis (AC) is an inflammatory disease of the gall bladder, and >90% of AC cases are associated with gallstones [1]. Cholecystitis represents one of the most common emergency admissions in surgical practice [2].

Cholecystectomy has been the gold standard treatment for AC. With the advent of laparoscopic cholecystectomy, early surgery is considered safe and cost effective for the management of AC [2]. However, in the elderly and patients with significant comorbidities, early cholecystectomy could result in morbidity up to 41% and perioperative mortality up to 18% [3–7].

Initial non-operative treatment, including antibiotic treatment with or without percutaneous cholecystostomy tube (PCT), is proposed for high-risk patients to prevent perioperative morbidity [8]. With the placement of PCT, the gallbladder is decompressed until the inflammatory process has subsided. Although interval cholecystectomy (IC) at 6–8 weeks after resolution of the initial AC is recommended [2, 9], some researchers have suggested that PCT may serve as a definitive treatment for AC in these high-risk patients who are unfit for surgery [10].

In the past decade, it has also been noted that PCT is increasingly used in less morbid patients [11]. For these patients, debate continues whether subsequent cholecystectomy is necessary. Knowledge of the prognosis for AC patients after PCT treatment may aid clinicians in the decision making for surgical intervention. This study was conducted retrospectively with an aim to estimate the recurrence rate in all adult AC patients after successful PCT treatment. The factors associated with recurrence were also investigated.

## Methods

### Study Setting and Patient Identification

We conducted a retrospective cohort study in a tertiary medical center, National Taiwan University Hospital (NTUH). Before data collection, the Institutional Review Board of the NTUH approved this study (reference number: 201401100RIN) and waived the requirement for informed consent because the current study was retrospective in design. NTUH is a 2 600-bed urban medical center providing all levels of care. Patients with AC were identified using the International Classification of Diseases, 9^th^ Revision, clinical modification codes 574.0, 574.3, 574.6, 574.8, 575.0, 575.12, and 575.4. Acute cholecystitis was diagnosed through a combination of patient history, physical examination, and laboratory analysis, as suggested by the Tokyo guidelines [12]. The diagnosis must be confirmed by characteristic imaging findings on ultrasonography (US) or computed tomography (CT) [12].

### Patient Management

Patients diagnosed with AC were kept nil per os (NPO), given sufficient infusion and electrolyte correction, and received antibiotics and analgesics. Consulting surgeons discussed risk-benefit profiles of early surgery, either laparoscopic or open cholecystectomy, with patients and/or their family members to achieve consensus regarding the final management strategy. Indications for PCT were based on surgeon discretion, which might include patient preference, failure of response to initial medical management, impending rupture of a severely distended gallbladder, and/or severe sepsis/septic shock.

Placement of PCTs was accomplished under local anesthesia using US or CT guidance at the discretion of the interventional radiologist. Fluoroscopy was used to confirm guidewire placement and the Seldinger technique was used to place 6 to 8 French pigtail catheters. A US-guided transhepatic approach through the right lobe was used to access the gallbladder.

After the resolution of AC, the PCT was removed if biliary symptoms did not recur after the PCT was temporarily clamped or if the cystic duct was patent on a formal cholangiography [13]. In contrast, if the risk of recurrence was expected to be high through the above assessments, the PCT might be left in situ until removed during cholecystectomy.

### Patient Selection and Data Collection

We used the following inclusion criteria: (1) adult AC patients admitted through the emergency department from October 1, 2004 through December 30, 2013; (2) absence of choledocholithiasis, hepatobiliary malignancy, or concurrent pancreatitis when AC was diagnosed; (3) patients receiving successful PCT treatment, which denoted patients surviving without the need for persistent PCT drainage.

The basic demographics, presenting vital signs, chief symptoms (fever or abdominal pain), physical findings (including right upper quadrant tenderness and Murphy’s sign), laboratory data (including white blood cell [WBC] count, C-reactive protein, and bilirubin levels), imaging findings (including gall bladder wall thickening, the presence of gallstones, distention of the gall bladder, and surrounding fluid accumulation) on US/CT, and the timing of PCT placement/removal were recorded. The severity of AC was graded according to the Tokyo guidelines [12]. Comorbidities were recorded according to the Charlson comorbidity index [14]. Gangrenous cholecystitis, emphysematous cholecystitis, gall bladder perforation, empyema, and pericholecystic abscess were categorized as complicated cholecystitis [12].

The primary outcome measure was recurrence of AC within one year and two months [15] after successful PCT treatment. The secondary outcome measure was IC within one year and two months [2, 9] after successful PCT treatment. Medical records of all patients were reviewed until December 2014.

### Statistical Analysis

We used R 2.15.3 software (R Foundation for Statistical Computing, Vienna, Austria) for data analysis. Categorical data were expressed as counts and proportions; continuous data were expressed as means and standard deviations. Categorical variables were compared by the Fisher’s exact test, and continuous variables were examined by the Wilcoxon rank-sum test. A two-tailed *P*-value of ≤0.05 was considered statistically significant.

We selected the odds ratio (OR) as the outcome measure. We conducted multivariable logistic regression analyses sequentially to examine the association between independent variables and outcomes, in the order of death, IC and then recurrence. Patients with the former outcome were excluded during the analysis of the next outcome. All available variables were considered in the regression model, regardless of whether they were significant by univariate analysis. The stepwise variable selection procedure (with iterations between the forward and backward steps) was applied to obtain the final regression model. Significance levels for entry and for stay were set at 0.15 to avoid exclusion of potential candidate variables. The final regression model was identified by excluding individual variables with a *P*-value >0.05, until all regression coefficients were statistically significant.

We used generalized additive models to examine the nonlinear effects of continuous variables and, if necessary, to identify the appropriate cut-off point(s) for dichotomizing a continuous variable during the variable selection procedure. We assessed the goodness-of-fit of the fitted regression model using C-statistics, adjusted generalized R^2^, and the Hosmer-Lemeshow test.

## Results

**Fig 1:**
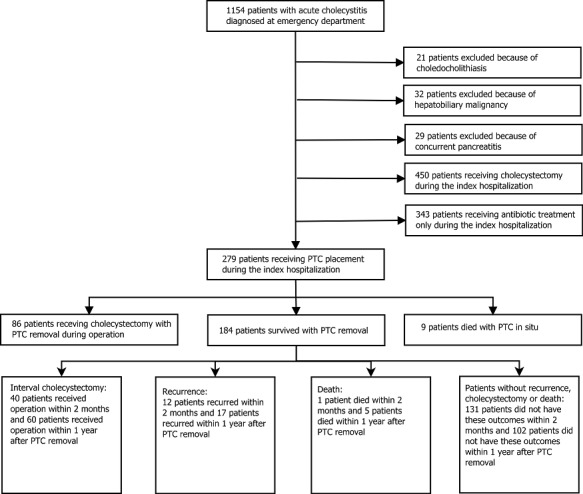
Patient enrollment flowchart. PCT, percutaneous cholecystostomy tube.

As shown in Fig 1, a total of 1 154 patients with AC were admitted through the emergency department from October 2004 through December 2013. Of these, 82 patients with choledocholithiasis, hepatobiliary malignancy, or concurrent pancreatitis were excluded. Of the remaining 1 072 patients, 450 underwent cholecystectomy, 343 received antibiotic treatment and 279 patients received PCT placement during the index hospitalization. Of these 279 patients, 9 (3.2%) patients died despite PCT placement; 86 patients (30.8%) had PCT left in situ and removed during cholecystectomy; the remaining 184 patients (65.9%) who completed successful PCT treatment and were free of persistent PCT drainage, were further assessed in the current analysis.

Table 1 summarizes the clinical characteristics and outcomes of the patients included in the study. There were 114 male patients (62%) and the mean age was 70.1 years. There were 73 patients (39.7%) who presented at the emergency department after symptom onset ≧3 days and 74 patients (40.2%) who presented with sepsis. Most of the patients (169/184, 91.8%) underwent CT imaging. A total of 138 (75.0%) patients had gallstones or sludge detected by US or CT and 41 patients (22.3%) suffered from complicated cholecystitis. There were 77 patients (41.8%) categorized as severity grade II per the Tokyo guidelines and 14 patients (7.6%) categorized as AC of severity grade III [12].

**Table 1:** Clinical Characteristics and Outcomes of Enrolled Patients.

Characteristics	All patients (n = 184)
Age, years (SD^*^)	70.1 (12.5)
Male, n (%)	114 (62.0)
Comorbidity, n (%)	
Diabetes mellitus	70 (38.0)
Cerebral vascular disease	36 (19.6)
Myocardial infarction	7 (3.8)
Congestive heart failure	8 (4.3)
Cirrhosis	8 (4.3)
Malignancy	23 (12.5)
Charlson comorbidity index (SD)	1.4 (1.6)
Bedridden status, n (%)	9 (4.9)
Previous abdominal surgery, n (%)	43 (23.4)
Clinical symptoms and signs on presentation at emergency department	
Abdominal pain, n (%)	138 (75.0)
Onset of symptoms before presentation, days (SD)	2.5 (2.9)
Onset of symptoms before presentation ≧3 days, n (%)	73 (39.7)
Body temperature, °C (SD)	37.3 (1.0)
Body temperature ≧38° C, n (%)	46 (25.0)
Mean arterial pressure, mm Hg (SD)	99.1 (20.0)
Mean arterial pressure ≦60 mmHg, n (%)	6 (3.3)
White blood cell count, 10^3^/μL (SD)	12.9 (5.5)
White blood cell count ≧18 000/μL, n (%)	26 (14.1)
Sepsis, n (%)	74 (40.2)
Diagnostic tools, n (%)	
Ultrasonography	77 (41.8)
Computed tomography	169 (91.8)
Findings on ultrasonography or computed tomography, n (%)	
Gall bladder stones or sludge	138 (75.0)
Complicated cholecystitis	41 (22.3)
Severity grade by Tokyo guidelines, n (%)	
Grade I	93 (50.5)
Grade II	77 (41.8)
Grade III	14 (7.6)
Early operation not suggested by surgeons, n (%)	105 (57.1)
Early operation rejected by patients, n (%)	53 (28.8)
Duration after presentation at emergency department, days (SD)	
Parenteral antibiotics	14.4 (8.9)
Fever	1.8 (2.0)
Parenteral analgesic use	1.2 (1.4)
Nil per os (NPO)	3.3 (2.3)
Hospital stay	17.5 (10.2)
PCT^†^ drainage	20.0 (25.7)
Outcomes, n (%)	
Events occurring within two months of PCT removal	
Two-month recurrence	12 (6.5)
Two-month cholecystectomy	40 (21.7)
Two-month death	1 (0.5)
Events occurring within one year of PCT removal	
One-year recurrence	17 (9.2)
One-year cholecystectomy	60 (32.6)
One-year death	(2.7)

* SD, standard deviation

† PCT, percutaneous cholecystostomy tube

Of the 184 patients, 77 (41.8%) were considered unsuitable for early cholecystectomy after surgical consultation; 53 patients (28.8%) rejected an offer of early cholecystectomy after discussion with surgeons; for the remaining patients, there was no explicit documentation. The average duration was 14.4 days for parenteral antibiotics and 20.0 days for PCT drainage. The one-year recurrence rate was 9.2% (17/184) with most recurrences within two months (6.5%, 12/184) after successful PCT treatment.

Because the number of deaths was small, these patients were excluded from comparison and further regression analysis. Table 2 presents the comparison between patients of one-year cholecystectomy with the rest of patients who did not experience death or IC. After exclusion of patients of one-year cholecystectomy, Table 3 presents the difference between patients with one-year recurrence and the remaining patients who did not experience death, IC, or recurrence. As shown in Table 4, age <70 years (OR: 4.00; 95% CI: 2.02–8.22; *P* < 0.001) and cerebral vascular disease (OR: 0.25; 95% CI: 0.07–0.72; *P* = 0.02) were positively and inversely associated with one-year cholecystectomy, respectively. For the primary outcome, complicated cholecystitis (OR: 4.67; 95% CI: 1.44–15.70; *P* = 0.01) and PCT drainage duration >32 days (OR: 4.92; 95% CI: 1.03–23.53; *P* = 0.05) positively correlated with one-year recurrence; parenteral antibiotics duration >10 days (OR: 0.21; 95% CI: 0.05–0.68; *p* = 0.01) was inversely associated with one-year recurrence.

**Table 2:** Clinical Characteristics Stratified by Outcome of One-Year Cholecystectomy.

Characteristics	Patients with one-year cholecystectomy (n = 60)	Patients without one-year cholecystectomy or death (n = 119)	*P* value
Age, years (SD*)	64.5 (14.1)	73.2 (15.5)	<0.001
Male, n (%)	35 (58.3)	76 (63.9)	0.516
Comorbidity, n (%)			
Diabetes mellitus	22 (36.7)	46 (38.7)	0.871
Cerebral vascular disease	4 (6.7)	31 (26.1)	0.002
Myocardial infarction	0 (0)	6 (5.0)	0.181
Congestive heart failure	0 (0)	7 (5.9)	0.097
Cirrhosis	3 (5.0)	5 (4.2)	1
Malignancy	5 (8.3)	16 (13.4)	0.461
Charlson comorbidity index (SD)	0.92 (1.2)	1.6 (1.6)	0.005
Bedridden status, n (%)	0 (0)	8 (6.7)	0.053
Previous abdominal surgery, n (%)	15 (25.0)	27 (22.7)	0.714
Clinical symptoms and signs on presentation at emergency department			
Abdominal pain, n (%)	47 (78.3)	87 (73.1)	0.473
Onset of symptoms before presentation, days (SD)	3.1 (3.4)	2.3 (2.6)	0.043
Onset of symptoms before presentation ≧3 days, n (%)	30 (50.0)	43 (36.1)	0.079
Body temperature, °C (SD)	37.1 (0.9)	37.3 (1.0)	0.189
Body temperature ≧38° C, n (%)	13 (21.7)	32 (26.9)	0.473
Mean arterial pressure, mm Hg (SD)	101.6 (24.1)	98.0 (17.7)	0.531
Mean arterial pressure ≦60 mm Hg, n (%)	2 (3.3)	4 (3.4)	1
White blood cell count, 10^3^/μL (SD)	12.3 (5.6)	13.2 (5.5)	0.194
White blood cell count ≧18 000/μL, n (%)	8 (13.3)	18 (15.1)	0.825
Sepsis, n (%)	21 (35.0)	51 (42.9)	0.337
Diagnostic tools, n (%)			
Ultrasonography	23 (38.3)	53 (44.5)	0.522
Computed tomography	55 (91.7)	109 (91.6)	1
Findings on ultrasonography or computed tomography, n (%)			
Gall bladder stones or sludge	47 (78.3)	87 (73.1)	0.473
Complicated cholecystitis	13 (21.7)	27 (22.7)	1
Severity grade by Tokyo guidelines, n (%)			
Grade I	31 (51.7)	58 (48.7)	0.753
Grade II	29 (48.3)	47 (39.5)	0.267
Grade III	0 (0)	14 (11.8)	0.003
Early operation not suggested by surgeons, n (%)	37 (61.7)	65 (54.6)	0.425
Early operation rejected by patients, n (%)	17 (28.3)	34 (28.6)	1
Duration after presentation at emergency department, days (SD)			
Parenteral antibiotics	13.5 (7.0)	14.9 (9.9)	0.260
Fever	2.0 (2.0)	1.8 (2.0)	0.385
Parenteral analgesic use	1.3 (1.2)	1.1 (1.5)	0.079
Nil per os (NPO)	3.1 (2.8)	3.3 (2.3)	0.511
Hospital stay	17.0 (10.5)	17.7 (10.2)	0.351
PCT^†^ drainage	17.0 (14.2)	20.9 (29.9)	0.647

* SD, standard deviation

† PCT, percutaneous cholecystostomy tube

**Table 3:** Clinical Characteristics Stratified by Outcome of One-Year recurrence.

Characteristics	Patients with one-year recurrence (n = 17)	Patients without one-year recurrence, cholecystectomy or death (n = 102)	*P* value
Age, years (SD*)	75.8 (12.6)	72.7 (15.9)	0.464
Male, n (%)	13 (76.5)	63 (61.8)	0.287
Comorbidity, n (%)			
Diabetes mellitus	7 (41.2)	39 (38.2)	0.796
Cerebral vascular disease	4 (23.5)	27 (26.5)	1
Myocardial infarction	1 (5.9)	5 (4.9)	1
Congestive heart failure	1 (5.9)	6 (5.9)	1
Cirrhosis	1 (5.9)	4 (3.9)	0.544
Malignancy	3 (17.6)	13 (12.7)	0.699
Charlson comorbidity index (SD)	1.6 (1.4)	1.6 (1.6)	0.614
Bedridden status, n (%)	1 (5.9)	7 (6.9)	1
Previous abdominal surgery, n (%)	9 (52.9)	18 (17.6)	0.003
Clinical symptoms and signs on presentation at emergency department			
Abdominal pain, n (%)	13 (76.5)	74 (72.5)	1
Onset of symptoms before presentation, days (SD)	2.8 (3.1)	2.2 (2.5)	0.572
Onset of symptoms before presentation ≧3 days, n (%)	8 (47.1)	35 (34.3)	0.414
Body temperature, °C (SD)	37.2 (0.8)	37.4 (1.1)	0.543
Body temperature ≧38°C, n (%)	1 (5.9)	31 (30.4)	0.039
Mean arterial pressure, mm Hg (SD)	99.1 (20.8)	97.8 (17.3)	0.912
Mean arterial pressure ≦60 mm Hg, n (%)	1 (5.9)	3 (2.9)	0.465
White blood cell count, 10^3^/μL (SD)	14.0 (5.6)	13.1 (5.5)	0.341
White blood cell count ≧18 000/μL, n (%)	4 (23.5)	14 (13.7)	0.287
Sepsis, n (%)	6 (35.3)	45 (44.1)	0.601
Diagnostic tools, n (%)			
Ultrasonography	8 (47.1)	45 (44.1)	1
Computed tomography	17 (100.0)	92 (90.2)	0.354
Findings on ultrasonography or computed tomography, n (%)			
Gall bladder stones or sludge	13 (76.5)	74 (72.5)	1
Complicated cholecystitis	8 (47.1)	19 (18.6)	0.024
Severity grade by Tokyo guidelines, n (%)			
Grade I	6 (35.3)	52 (51.0)	0.298
Grade II	9 (52.9)	38 (37.3)	0.285
Grade III	2 (11.8)	12 (11.8)	1
Early operation not suggested by surgeons, n (%)	7 (41.2)	58 (56.9)	0.295
Early operation rejected by patients, n (%)	6 (35.3)	28 (27.5)	0.565
Duration after presentation at emergency department, days (SD)			
Parenteral antibiotics	12.6 (7.2)	15.2 (10.2)	0.148
Fever	1.4 (2.3)	1.8 (2.0)	0.142
Parenteral analgesic use	0.8 (1.0)	1.1 (1.6)	0.628
Nil per os (NPO)	4.1 (2.7)	3.2 (2.2)	0.135
Hospital stay	14.7 (7.4)	18.3 (10.6)	0.129
PCT^†^ drainage	36.8 (68.4)	18.3 (15.8)	0.233

* SD, standard deviation

† PCT, percutaneous cholecystostomy tube

**Table 4:** Multiple Logistic Regression Model with One-Year Outcome as the Dependent Variable.

Independent variable^†^	Odds ratio	95% confidence interval	*P* value^†^
*Outcome*: *One-year cholecystectomy*^‡^			
Age <70 years	4.00	2.02–8.22	<0.001
Cerebral vascular disease	0.25	0.07–0.72	0.018
*Outcome*: *One-year recurrence*^§^			
Complicated cholecystitis	4.67	1.44–15.70	0.010
Parenteral antibiotics duration >10 days	0.21	0.05–0.68	0.013
PCT* drainage duration >32 days	4.92	1.03–23.53	0.046

† The display of independent variables is arranged in order of *P* value

‡ For model of one-year cholecystectomy: Goodness-of-fit assessment: n = 179; adjusted generalized *R*^*2*^ = 0.206; the estimated area under the receiver operating characteristic (ROC) curve = 0.725; and modified Hosmer-Lemeshow F test *P* value = 0.17.

§ For model of one-year recurrence: Goodness-of-fit assessment: n = 119; adjusted generalized *R*^*2*^ = 0.206; the estimated area under the receiver operating characteristic (ROC) curve = 0.742; and modified Hosmer-Lemeshow F test *P* value = 0.92.

* PCT, percutaneous cholecystostomy tube

The comparisons of two-month outcomes are presented in S1 Table and S2 Table. As shown in Table 5, the factors associated with two-month outcomes were similar to those associated with one-year outcomes.

**Table 5:** Multiple Logistic Regression Model with Two-Month Outcomes as the Dependent Variable.

Independent variable*	Odds ratio	95% confidence interval	*P* value*
*Outcome*: *Two-month cholecystectomy*^†^			
Age <70 years	3.84	1.76–8.75	< 0.001
Grade II by Tokyo guidelines	3.04	1.41–6.80	0.005
Charlson comorbidity index	0.68	0.48–0.91	0.018
*Outcome*: *Two-month recurrence*^‡^			
Complicated cholecystitis	5.94	1.57–23.96	0.009
White blood cell count ≧18 000/μL	5.16	1.14–22.34	0.027
Parenteral antibiotics duration >10 days	0.25	0.06–0.92	0.042

* The display of independent variables is arranged in order of *P* value

† For model of two-month cholecystectomy: Goodness-of-fit assessment: n = 183; adjusted generalized *R*^*2*^ = 0.224; the estimated area under the receiver operating characteristic (ROC) curve = 0.767; and modified Hosmer-Lemeshow F test *P* value = 0.97.

‡ For model of two-month recurrence: goodness-of-fit assessment: n = 143; adjusted generalized *R*^*2*^ = 0.236; the estimated area under the receiver operating characteristic (ROC) curve = 0.788; and modified Hosmer-Lemeshow F test *P* value = 0.97.

## Discussion

In this retrospective observational study, the results of a decade of experience showed that the recurrence rate after successful PCT treatment was low: 6.5% (12/184) occurring within 2 months and 9.2% (17/184) occurring within one year after treatment. The analysis also indicated that the risk factors associated with increased recurrence were the severity of AC per se, including complicated cholecystitis and WBC counts >18 000/μL, and offered treatments, including duration of antibiotics and PCT treatment.

The PCT technique was introduced by Radder [16] in1980, and has been established as a cost-effective and reliable procedure for high-risk patients [17]. The procedure-related mortality rate was <3% [17, 18]. Studies have indicated that >80% of patients experienced rapid relief from the clinical symptoms of AC within 3 days after PCT placement [18–21]. After resolution of AC, it was reported to be desirable to perform IC to prevent recurrence [22]. Nevertheless, an increasing number of studies have indicated that PCT might not only serve as a bridge to IC but could probably be a definitive treatment for AC, especially for patients with high operative risk [23–25].

For patients with less comorbidity, the necessity of subsequent IC remained more contentious. It has been reported that while PCT was often performed with the intent of IC, less than half of patients actually underwent surgery after PCT [26]. Elucidating the trajectory after successful PCT treatment might help patients and clinicians in the formulation of optimal management strategy. However, there is a lack of a clear definition for “successful PCT treatment.” Whether patients with resolution of AC but with PCT left in situ until removed during cholecystectomy should be counted as “successful PCT treatment” remains undefined. The result might be biased if these patients were pooled in analysis because these patients were not at risk for recurrence due to the persistent drainage by PCT. Therefore, in our study, we defined that only patients free of persistent PCT drainage were those treated successfully by PCT and observed for recurrence.

The recurrence rate after PCT placement ranged from 4% to 22% [8, 23, 27, 28]. In our study, the one-year recurrence rate was relatively low, 17/184 (9.2%), with most recurrences within two months (12/184, 6.5%) after successful PCT treatment. This low recurrence rate might be explained by two reasons. First, patients considered high for recurrence was discharged with PCT in situ until cholecystectomy (86/279; 30.8%) (Fig 1). In our hospital, the PCT could usually be removed after temporary clamping of the drain had been shown to be well-tolerated. Some clinicians favored performing a cholangiography via the drain before withdrawal to ensure the absence of leakage or an obstructed cystic duct, but this policy was not systematic [13]. Second, some of the patients after successful PCT treatment received IC within one year (60/184; 32.6%) (Fig 1). Clearly, these patients receiving IC would not be susceptible to recurrence. Including these patients in the denominator for calculation might underestimate the actual recurrence rate. Nevertheless, excluding these patients in the rate calculation would also make it difficult for clinicians to apply the analysis results to predict the prognosis of an incoming patient without knowing in advance whether he/she would receive IC within one year.

There have been no recommendations proposed for the optimal duration of PCT drainage. The duration of drainage ranged from three to six weeks, one month on the average, in previous investigations [8]. In our cohort, the mean PCT drainage duration was 20 days. According to previous reports, ≧2 weeks were required for tract maturation for the transhepatic approach and 3 weeks were required for the transperitoneal approach [29, 30]. Nevertheless, the result showed that the PCT drainage duration longer than one month was associated with one-year recurrence (OR: 4.92). Few studies examined the association between drainage duration with recurrence. Hsieh et al. [15] indicated that a drainage duration >2 weeks was associated with increased recurrence within two months of the initial AC attack, probably caused by irritation of the gallbladder mucosa by the PCT [31, 32] or bacterial colonization of the tube [33]. While these theories [31–33] might help explain increased short-term recurrence, they might not account for increased one-year recurrence. In our study, patients whose PCT could not be removed earlier might be those who could barely tolerate temporary clamping of PCT or had an obstructed cystic duct on cholangiography, which suggested that these patients might have a higher recurrence probability. Therefore, we suggested that for patients who needed PCT drainage longer than one month should have PCT kept in situ until cholecystectomy if possible. This suggestion might be contradictory to the result of the study by Hsieh et al. [15]. However, Hsieh et al. [14] did not explicitly reveal the timing and the risk stratification method for PCT removal, which made it difficult to compare the results of the current analysis to that of Hsieh et al. [15].

Currently, there are no specific recommendations for antibiotic therapy in association with PCT drainage. In our previous study [34], we noted that in patients receiving antibiotic therapy alone for AC, a duration of parenteral antibiotic use >8 days was associated with decreased recurrence. The Tokyo guidelines [35] suggested for AC of grade II and III, 4–7 days of antibiotic administration was recommended. Nevertheless, the developers of the guidelines [35] also admitted that there were very few data available for the treatment duration of AC and their recommendations [35] referred to the duration of antibiotic therapy for complicated intraabdominal infections suggested by SIS-NA/IDSA 2010 guidelines [36]. In the current analysis, we noted that the duration of parenteral antibiotic use >10 days was associated with both decreased one-year and two-month recurrence, which might serve as a reference for the duration of antibiotic administration for patients requiring PCT drainage. Nevertheless, although we controlled most clinical and laboratory variables in our model, we might still overlook some important variables to eliminate the bias of confounding by indication. Furthermore, the administered antibiotics in the current analysis were heterogeneous, which might also limit the applicability of this result.

Complicated cholecystitis and elevated WBC counts ≧18 000/μL were noted to correlate with an increased risk of recurrence. Both factors were used to define AC of severity grade II in the Tokyo guidelines [12] because the presence of these factors suggested severe gallbladder inflammation, which might be associated with increased operative difficulty when performing early cholecystectomy. For these patients with AC of grade II, the Tokyo guidelines [22] suggested IC be performed after the improvement of the acute inflammatory process. Our analysis demonstrated that patients with AC of grade II were indeed more likely to receive IC within two months after successful PCT treatment (OR: 3.04), as recommended by the guidelines [22]; in contrast, if they did not receive IC, they were also more likely to suffer a two-month recurrence (complicated cholecystitis, OR: 5.94; WBC count ≧18 000/μL; OR: 5.16). This information might be important to corroborate the necessity of IC in these patients. However, as the regression analysis indicated, age and comorbidities were still important surgical considerations for IC. For patients with old age or advanced comorbidities suffering from AC of grade II, the uncertainties remained about whether they should receive IC to prevent recurrence or receive repeated PCT drainage to avoid perioperative morbidity.

In our study, we used multivariable regression analysis sequentially to identify independent factors significantly associated with each outcome. We could have conducted a survival analysis by fitting a Cox’s proportional hazards model. Nevertheless, because the duration of PCT drainage might correlate with the observation duration for each outcome, in violation of the independent censoring assumption required by a Cox’s proportional hazards model, we decided to adopt the current analysis method.

The efficacy of PCT treatment in high-risk patients has been acknowledged by previous studies [23–25]. For these high-risk patients, even if they had been medically optimized, they might still suffer from significant perioperative comorbidities if they agreed to receive IC [3–7]. Therefore, it might be less debatable that PCT could serve as a definitive treatment for them. Nevertheless, it was noted that there was a trend toward PCT being increasingly used in less morbid patients [11]. For these patients, a complete benefit-risk profile might be important for them to consider whether elective cholecystectomy was mandatory because our analysis showed that even if faced with emergent conditions, up to 28.8% (53/184) of patients still did not want to undergo early cholecystectomy. Our study addressed some concerns for these less morbid patients; nevertheless, only a prospective study like the ongoing CHOCOLATE trial [37], could illustrate the complete risk-benefit picture for them.

### Limitations

First, this was an observational study and as such, we were only able to establish an association, rather than a causal relationship, between independent and outcome variables. Second, although our hospital provided all levels of care, the cohort from a single medical center may still introduce selection bias. Third, the recurrence rate in our study might be underestimated because we could not exclude the possibility that patients would receive treatment for recurrence at other hospitals. However, all patients visited our emergency department at the time of initial AC, suggesting that many might utilize the same emergency medical services again in the case of recurrence. This might compensate to some extent for the underestimation. Also, this kind of misclassification bias usually leads to an underestimated association between independent and dependent variables, which might be a less problem for those identified statistically significant independent variables

## Conclusions

The recurrence rate after successful PCT treatment was low. Patients with complicated cholecystitis, elevated WBC counts, or need for prolonged PCT drainage were more likely to experience recurrence.

## Supporting Information

S1 Dataset Raw data used in statistical analysis. (XLSX)

S1 Table Clinical Characteristics Stratified by Outcome of Two-Month Cholecystectomy. (DOCX)

S2 Table Clinical Characteristics Stratified by Outcome of Two-Month Recurrence. (DOCX)
