# Supplementary material for: Long-Term Outcomes of Patients with Acute Cholecystitis after Successful Percutaneous Cholecystostomy Treatment and the Risk Factors for Recurrence: A Decade Experience at a Single Center
Source: PLoS One. 2016 Jan 28;11(1):e0148017. doi: 10.1371/journal.pone.0148017 (PMC4731150; doi:10.1371/journal.pone.0148017)
Supplement: S1 Table — (DOCX) [file pone.0148017.s002.docx]

**S1 Table.** Clinical Characteristics Stratified by Outcome of Two-Month Cholecystectomy

| Characteristics | Patients with two-month cholecystectomy  (n = 40) | Patients without two-month cholecystectomy or death(n = 143) | *P* value |
| --- | --- | --- | --- |
| Age, years (SD^*^) | 64.3 (14.7) | 71.8 (15.5) | 0.003 |
| Male, n (%) | 24 (60.0) | 89 (62.2) | 0.855 |
| Comorbidity, n (%) |  |  |  |
| Diabetes mellitus | 13 (32.5) | 56 (39.2) | 0.468 |
| Cerebral vascular disease | 2 (5.0) | 34 (23.8) | 0.007 |
| Myocardial infarction | 0 (0) | 7 (4.9) | 0.350 |
| Congestive heart failure | 0 (0) | 8 (5.6) | 0.204 |
| Cirrhosis | 1 (2.5) | 7 (4.9) | 1 |
| Malignancy | 2 (5.0) | 20 (14.0) | 0.170 |
| Charlson comorbidity index (SD) | 0.8 (1.0) | 1.6 (1.6) | 0.003 |
| Bedridden status, n (%) | 0 (0) | 9 (6.3) | 0.209 |
| Previous abdominal surgery, n (%) | 10 (25.0) | 33 (23.1) | 0.834 |
| Clinical symptoms and signs on presentation at emergency department |  |  |  |
| Abdominal pain, n (%) | 31 (77.5) | 106 (74.1) | 0.837 |
| Onset of symptoms before presentation, days (SD) | 3.4 (4.0) | 2.3 (2.5) | 0.089 |
| Onset of symptoms before presentation ≧3 days, n (%) | 19 (47.5) | 54 (37.8) | 0.279 |
| Body temperature, °C (SD) | 37.2 (1.0) | 37.3 (1.0) | 0.619 |
| Body temperature ≧38° C, n (%) | 11 (27.5) | 35 (24.5) | 0.685 |
| Mean arterial pressure, mm Hg (SD) | 98.8 (24.9) | 99.1 (18.5) | 0.762 |
| Mean arterial pressure ≦60 mm Hg, n (%) | 2 (5.0) | 4 (2.8) | 0.613 |
| White blood cell count, 10^3^/µL (SD) | 13.0 (6.2) | 12.9 (5.3) | 0.797 |
| White blood cell count ≧18 000/µL, n (%) | 7 (17.5) | 19 (13.3) | 0.608 |
| Sepsis, n (%) | 17 (42.5) | 57 (39.9) | 0.856 |
| Diagnostic tools, n (%) |  |  |  |
| Ultrasonography | 13 (32.5) | 63 (44.1) | 0.209 |
| Computed tomography | 36 (90.0) | 132 (92.3) | 0.744 |
| Findings on ultrasonography or computed tomography, n (%) |  |  |  |
| Gall bladder stones or sludge | 31 (77.5) | 106 (74.1) | 0.837 |
| Complicated cholecystitis | 10 (25.0) | 31 (21.7) | 0.671 |
| Severity grade by Tokyo guidelines, n (%) |  |  |  |
| Grade I | 16 (40.0) | 76 (53.1) | 0.156 |
| Grade II | 24 (60.0) | 53 (37.1) | 0.011 |
| Grade III | 0 (0) | 14 (9.8) | 0.042 |
| Early operation not suggested by surgeons, n (%) | 26 (65.0) | 79 (55.2) | 0.285 |
| Early operation rejected by patients, n (%) | 10 (25.0) | 42 (29.4) | 0.693 |
| Duration after presentation at emergency department, days (SD) |  |  |  |
| Parenteral antibiotics | 15.0 (7.1) | 14.3 (9.4) | 0.387 |
| Fever | 2.2 (2.1) | 1.7 (2.0) | 0.167 |
| Parenteral analgesic use | 1.4 (1.2) | 1.1 (1.5) | 0.075 |
| Nil per os (NPO) | 3.2 (2.4) | 3.3 (2.3) | 0.592 |
| Hospital stay | 18.6 (11.7) | 17.2 (9.8) | 0.616 |
| PCT^†^ drainage | 18.9 (16.4) | 20.0 (27.5) | 0.753 |

* SD, standard deviation

† PCT, percutaneous cholecystostomy tube
