# Supplementary material for: Long-Term Outcomes of Patients with Acute Cholecystitis after Successful Percutaneous Cholecystostomy Treatment and the Risk Factors for Recurrence: A Decade Experience at a Single Center
Source: PLoS One. 2016 Jan 28;11(1):e0148017. doi: 10.1371/journal.pone.0148017 (PMC4731150; doi:10.1371/journal.pone.0148017)
Supplement: S2 Table — (DOCX) [file pone.0148017.s003.docx]

**S2 Table.** Clinical Characteristics Stratified by Outcome of Two-Month Recurrence

| Characteristics | Patients with two-month recurrence  (n = 12) | Patients without two-month recurrence, cholecystectomy or death (n = 131) | *p* value |
| --- | --- | --- | --- |
| Age, years (SD^*^) | 75.9 (9.6) | 71.4 (15.9) | 0.417 |
| Male, n (%) | 10 (83.3) | 79 (60.3) | 0.134 |
| Comorbidity, n (%) |  |  |  |
| Diabetes mellitus | 5 (41.7) | 51 (38.9) | 1 |
| Cerebral vascular disease | 3 (25.0) | 31 (23.7) | 1 |
| Myocardial infarction | 1 (8.3) | 6 (4.6) | 0.466 |
| Congestive heart failure | 1 (8.3) | 7 (5.3) | 0.513 |
| Cirrhosis | 1 (8.3) | 6 (4.6) | 0.466 |
| Malignancy | 3 (25.0) | 17 (13.0) | 0.375 |
| Charlson comorbidity index (SD) | 2.0 (1.4) | 1.5 (1.6) | 0.200 |
| Bedridden status, n (%) | 0 (0) | 9 (6.9) | 1 |
| Previous abdominal surgery, n (%) | 8 (66.7) | 25 (19.1) | 0.001 |
| Clinical symptoms and signs on presentation at emergency department |  |  |  |
| Abdominal pain, n (%) | 9 (75.0) | 97 (74.0) | 1 |
| Onset of symptoms before presentation, days (SD) | 3.2 (3.2) | 2.2 (2.4) | 0.260 |
| Onset of symptoms before presentation ≧3 days, n (%) | 7 (58.3) | 47 (35.9) | 0.211 |
| Body temperature, °C (SD) | 37.3 (0.9) | 37.3 (1.0) | 0.994 |
| Body temperature ≧38° C, n (%) | 1 (8.3) | 34 (26.0) | 0.294 |
| Mean arterial pressure, mm Hg (SD) | 100.7 (23.8) | 99.0 (18.1) | 0.782 |
| Mean arterial pressure ≦60 mm Hg, n (%) | 1 (8.3) | 3 (2.3) | 0.299 |
| White blood cell count, 10^3^/µL (SD) | 15.0 (5.8) | 12.7 (5.3) | 0.117 |
| White blood cell count ≧18 000/µL, n (%) | 4 (33.3) | 15 (11.5) | 0.056 |
| Sepsis, n (%) | 5 (41.7) | 52 (39.7) | 1 |
| Diagnostic tools, n (%) |  |  |  |
| Ultrasonography | 7 (58.3) | 56 (42.7) | 0.368 |
| Computed tomography | 12 (100.0) | 120 (91.6) | 0.599 |
| Findings on ultrasonography or computed tomography, n (%) |  |  |  |
| Gall bladder stones or sludge | 9 (75.0) | 97 (74.0) | 1 |
| Complicated cholecystitis | 6 (50.0) | 25 (19.1) | 0.023 |
| Severity grade by Tokyo guidelines, n (%) |  |  |  |
| Grade I | 3 (25.0) | 73 (55.7) | 0.067 |
| Grade II | 7 (58.3) | 46 (35.1) | 0.128 |
| Grade III | 2 (16.7) | 12 (9.2) | 0.333 |
| Early operation not suggested by surgeons, n (%) | 4 (33.3) | 75 (57.3) | 0.136 |
| Early operation rejected by patients, n (%) | 5 (41.7) | 37 (28.2) | 0.335 |
| Duration after presentation at emergency department, days (SD) |  |  |  |
| Parenteral antibiotics | 11.5 (5.8) | 14.5 (9.6) | 0.203 |
| Fever | 1.6 (2.6) | 1.7 (1.9) | 0.447 |
| Parenteral analgesic use | 0.7 (0.9) | 1.2 (1.5) | 0.342 |
| Nil per os (NPO) | 4.6 (3.0) | 3.2 (2.2) | 0.080 |
| Hospital stay | 14.0 (6.4) | 17.5 (10.0) | 0.238 |
| PCT^†^ drainage | 44.8 (80.5) | 17.7 (14.8) | 0.104 |

* SD, standard deviation

† PCT, percutaneous cholecystostomy tube
